# Higher Order Visual Location Learning Does Not Explain Multisensory Enhancement of Sound Localization (Reply to Vroomen and Stekelenburg 2021)

**DOI:** 10.1111/ejn.70132

**Published:** 2025-04-30

**Authors:** Patrick Bruns, Hubert R. Dinse, Brigitte Röder

**Affiliations:** ^1^ Biological Psychology and Neuropsychology University of Hamburg Hamburg Germany; ^2^ Neural Plasticity Lab, Institute of Neuroinformatics Ruhr University Bochum Bochum Germany; ^3^ LV Prasad Eye Institute Hyderabad India

**Keywords:** crossmodal learning, recalibration, spatial hearing, ventriloquism

## Abstract

In a recent study, we reported that multisensory enhancement (ME) of auditory localization after exposure to spatially congruent audiovisual stimuli and crossmodal recalibration in the ventriloquism aftereffect (VAE) are differently affected by the temporal stimulation frequency with which the audiovisual exposure stimuli are presented. Because audiovisual stimulation at 10 Hz rather than at 2 Hz selectively abolished the VAE but did not affect the ME, we concluded that distinct underlying neural mechanisms are involved in the two effects. A commentary on our paper challenged this interpretation and argued that the ME might have been spared simply because participants had acquired higher order knowledge about the loudspeaker locations from the visual stimulus locations in the ME condition, or because the ME was generally more reliable than the VAE. To test this alternative explanation of our results, we conducted an additional control experiment in which participants localized sounds before and after exposure to unimodal visual stimulation at the loudspeaker locations. No significant reduction of auditory localization errors was found after unimodal visual exposure, suggesting that higher order visual location learning cannot sufficiently explain the significant ME that was observed after audiovisual exposure in our previous study. These new results confirm previous findings pointing toward dissociable neural mechanisms underlying ME and VAE.

Perceptual processes remain highly adaptive throughout life. For example, in adult human participants, brief exposure to audiovisual stimuli results in an adjustment of subsequent unimodal sound localization. Depending on the spatial configuration of the audiovisual stimuli, crossmodal learning can either result in a reduction of sound localization errors if the audiovisual exposure stimuli are spatially congruent (multisensory enhancement, ME), or it can result in a recalibration of the perceived sound location toward the visual side if the audiovisual exposure stimuli feature a consistent spatial discrepancy (ventriloquism aftereffect, VAE). In a recent study (Bruns et al. 2020), we demonstrated that ME and VAE are differently affected by the temporal frequency with which the exposure stimuli are presented: Continuous audiovisual stimulation at a low frequency of two stimuli per second reliably induced both effects, whereas intermittent audiovisual stimulation at a high frequency of 10 Hz selectively abolished the VAE but did not affect the ME. We concluded that this dissociation between VAE and ME suggests an involvement of distinct underlying neural mechanisms in the two effects. The VAE likely depends on processing loops in cortical networks which may be too slow to integrate the spatial attributes of audiovisual stimuli at frequencies above 4 Hz (Bonath et al. 2007), whereas ME induced by spatially congruent stimuli might be mediated by subcortical processes that do not require a time‐consuming looping through distant neural circuits (Passamonti et al. 2009).

In a commentary on our paper, Vroomen and Stekelenburg (2021) raised the possibility that alternative and simpler explanations could account for the differential effect of temporal stimulation frequency on the VAE and ME, which would not necessarily require assuming dissociable underlying neural mechanisms. More specifically, they challenged our interpretation of the results on two accounts: (a) the VAE might generally be less reliable than the ME, and (b) the participants in our study might have simply acquired higher order knowledge about the loudspeaker locations from the visual stimulus locations. In this view, multisensory processing of the audiovisual stimuli was equally impaired by the high stimulation frequency, but only the VAE (and not the ME) subsided because it was more vulnerable and/or because participants could not resort to visually acquired location knowledge in this condition.

Based on the available evidence, we consider it as highly unlikely that the VAE indeed “declines faster, is easier to erase, requires more time to build up, or is just more difficult to measure” (Vroomen and Stekelenburg 2021, 3638) than the ME. The VAE has been replicated in numerous studies which have consistently shown that the effect builds up maximally within just a few minutes (Frissen et al. 2012) and can persist even across days (Bruns and Röder 2019), making it one of the most robust experimental paradigms in cognitive neuroscience research (for a recent review, see Bruns 2019). By contrast, studies of the ME are relatively scarce and sometimes reported improvements in sound localization only after extensive training over several days (Strelnikov et al. 2011). The manifest conclusion that the VAE is actually more reliable than the ME is also reflected in the results of our study (Bruns et al. 2020): The effect size of the standard VAE (*d* = 1.61) was much larger than the effect size of the ME (*d* = 0.64), and the evidence in favor of an effect that is larger than zero, as assessed with Bayesian statistics, was much stronger for the VAE (BF = 2244) than it was for the ME (BF = 5).

An influence of higher order knowledge about the loudspeaker locations, which could theoretically be derived solely from the visual locations and without a need to integrate the audiovisual exposure stimuli, might, however, be less easily dismissed. On the one hand, it seems disputable why participants should infer the loudspeaker locations from the visual locations only in the ME but not in the VAE condition, given that they are usually unaware of the audiovisual spatial disparity. On the other hand, previous studies have indeed shown that visual observation of the loudspeaker setup, even if available only briefly and prior to performing the sound localization task, can improve auditory spatial perception (Tonelli et al. 2015). It is, however, unclear whether simple unimodal visual stimuli, presented at the locations of the loudspeakers, would be sufficient to improve sound localization performance.

Previous studies of the VAE and ME have not investigated the influence of unimodal visual exposure on subsequent sound localization. We, therefore, tested this control condition in a sample of 14 healthy adults (mean age: 24.9 years; range: 20–38 years; 12 female and 2 male). As in our previous study (Bruns et al. 2020), participants used a hand pointer to indicate the perceived azimuthal location of 1000 Hz tones that were presented with a duration of 30 ms from six different loudspeaker locations spanning ± 22.5° in 9° increments (15 trials per location). The sound localization task was performed before and after a visual exposure phase, in which unimodal visual stimuli (red LEDs) were presented at the loudspeaker locations (100 trials per location). Visual stimuli were presented at a rate of two stimuli per second and changed locations after every tenth stimulus. Participants had to detect rare deviant stimuli (1% of the trials) with a longer duration (490 ms instead of 30 ms), but did not engage in an active localization task in this phase of the experiment. The experimental procedure was approved by the ethics commission of the German Psychological Society (DGPs) and the study was performed in accordance with the ethical standards laid down in the Declaration of Helsinki. All participants provided written informed consent prior to taking part in the study.

We had defined the ME as a significant reduction in the absolute localization error after exposure to spatially congruent audiovisual stimuli as compared to pretest (Bruns et al. 2020). In the present experiment, we did not observe a comparable significant error reduction after unimodal visual exposure (*M* = 0.7°, SEM = 1.0; *t*_13_ = 0.77, *p* = 0.455, *d* = 0.21; BF_10_ = 0.35), despite similar statistical power as in our previous study. As would be expected if visual location learning had no effect on subsequent auditory localization performance, absolute localization errors numerically increased in half of the participants, and numerically decreased in the other half (see Figure 1). Because the absolute error is a composite measure of localization accuracy and precision, we additionally tested for posttest changes in constant error (accuracy only) and in variable error (precision only). Constant errors numerically even increased from pretest to posttest (*M* = −0.5°, SEM = 0.8), but this change was not significantly different from zero (*t*_13_ = −0.66, *p* = 0.520, *d* = −0.18; BF_10_ = 0.33). By contrast, variable errors numerically decreased from pretest to posttest (*M* = 1.7°, SEM = 1.2), albeit not significantly either (*t*_13_ = 1.46, *p* = 0.168, *d* = 0.39; BF_10_ = 0.65). However, in 12 out of 14 participants, variable errors were numerically lower at posttest (see Figure 1), which is significantly more participants than would be expected by chance (binomial test: *p* = 0.013). Thus, if at all, unimodal visual exposure had an effect on sound localization precision, possibly by narrowing the overall range of likely target locations, but it clearly did not improve sound localization accuracy. Improved accuracy would, however, have been expected if participants had indeed obtained higher order knowledge about the exact loudspeaker locations.

**FIGURE 1:**
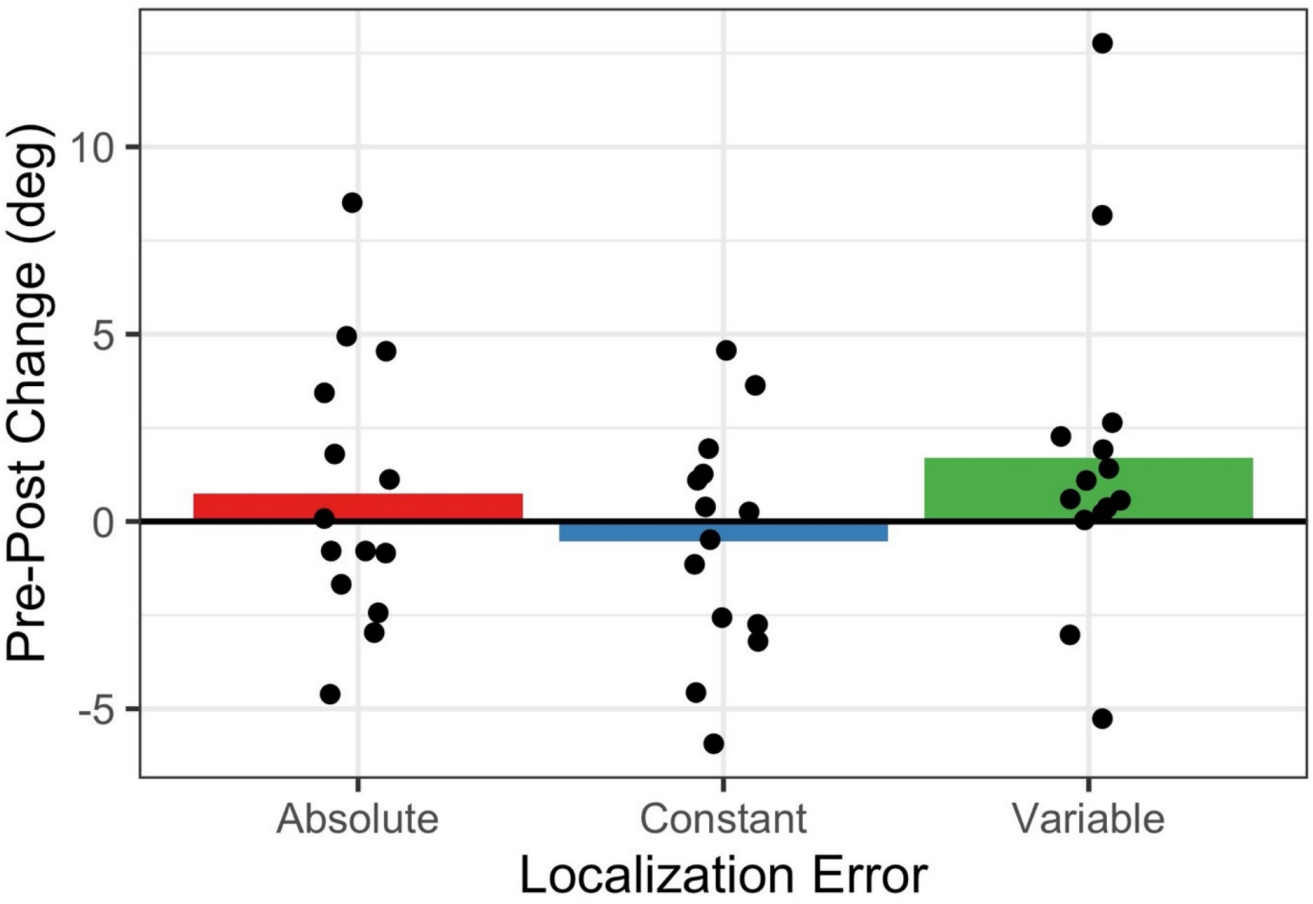
Changes in sound localization error after exposure to unimodal visual stimuli relative to baseline. Positive values indicate error reductions (i.e., better performance) and negative values indicate error increases (i.e., worse performance) at posttest. Bars indicate group means, and superimposed dots indicate individual values. Three types of error measures were considered: absolute errors (reflecting accuracy and precision), which were calculated by averaging the absolute values of the single‐trial localization errors; constant errors (reflecting accuracy only), which were calculated by averaging the signed single‐trial localization errors; and variable errors (reflecting precision only), which were calculated as the standard deviation of the single‐trial localization errors.

Taken together, the influence of visual exposure on sound localization was small at best and does not sufficiently explain the significant ME observed in the audiovisual exposure conditions of our previous study (Bruns et al. 2020). This suggests that the ME requires genuine multisensory integration processes, which, if identical to the ones underlying the VAE, should have been equally sensitive to the manipulation of temporal stimulation frequency as the VAE. Thus, any full‐fledged account of our result pattern would need to assume dissociable underlying learning mechanisms for the VAE and ME.

Neuropsychological studies in hemianopic patients have suggested the collicular–extrastriate pathway as a likely candidate for a neural mechanism that could underlie the spared ME in our high‐frequency stimulation condition (Lewald et al. 2012; Passamonti et al. 2009).

Ultimately, neuroimaging studies will be needed to verify this hypothesis.

## Author Contributions

**Patrick Bruns:** conceptualization, data curation, formal analysis, funding acquisition, investigation, methodology, visualization, writing – original draft, writing – review and editing. **Hubert R. Dinse:** conceptualization, writing – review and editing. **Brigitte Röder:** conceptualization, funding acquisition, resources, supervision, writing – review and editing.

## Conflicts of Interest

The authors declare no conflicts of interest.

### Peer Review

The peer review history for this article is available at https://www.webofscience.com/api/gateway/wos/peer‐review/10.1111/ejn.70132.

## Data Availability

The raw data associated with this study are publicly available in the research data repository of the University of Hamburg at https://doi.org/10.25592/uhhfdm.17417.
